# The Disease of Inebriety from Alcohol, Opium, and Other Narcotic Drugs

**Published:** 1893-05

**Authors:** 


The Disease of Inebriety from Alcohol, Opium, and Other Narcotic Drugs. Its Ætiology, Pathology, Treatment, and Medico-legal Relations, arranged and compiled by the American Association for the Study and Cure of Inebriety. New York : E. B. Treat, 1893. Price, $2.75.

This work is edited by Dr. T. D. Crothers, the Secretary of the American Association, whose writings on the subject of Inebriety as a disease demanding medical treatment are already known to the profession. The aim of the author is a volume that shall "contain the most reliable conclusions and studies of eminent authorities on the subject up to the present time." The work is intended as an introduction to the scientific study of the "drink malady," and gives general views of probable facts sustained by strong evidence. Some of the chapters are devoted to drug poisonings, as Morphia, Cocaine, Ether, etc. Chapter XXI, on the general considerations of treatment, and Chapter XXII, to asylums and their work. The volume in the whole is interesting and will prove a useful addition to the physician's library.

